# Acupressure in perinatal care: Results of a cross-sectional study in Malta

**DOI:** 10.18332/ejm/219644

**Published:** 2026-06-15

**Authors:** Chanelle Galea, Rita Pace Parascandalo

**Affiliations:** 1 Department of Midwifery, Faculty of Health Sciences, University of Malta, Msida, Malta

**Keywords:** midwives, acupressure, perinatal care, complementary therapies, knowledge and attitudes

## Abstract

**INTRODUCTION:**

Acupressure, the application of firm pressure to specific acupoints, has been associated with benefits during the perinatal period, including reducing nausea, alleviating pain, inducing labor, supporting lactation and decreasing anxiety. While research has explored its effectiveness, little is known about midwives’ knowledge and views regarding acupressure and its integration into practice. This study aimed to explore midwives’ knowledge, perspectives and factors influencing their use of acupressure during the perinatal period.

**METHODS:**

A quantitative, cross-sectional survey design was employed using a self-administered online and paper-based questionnaire. This was a total population study utilizing a non-probability sampling technique where the entire population of 240 midwife members of the Malta Midwives Association (MMA) was included. Eligible participants included practicing members in any maternity care setting across the Maltese Islands and these were invited to complete the survey. After conducting a pilot study, data collection took place between July and September 2024, where a total of 94 responses were received. Quantitative data were analyzed using descriptive and inferential statistics, while open-ended responses underwent thematic analysis.

**RESULTS:**

While midwives demonstrated a strong interest in acupressure, their hesitation to practice stemmed primarily from insufficient training (20.7%; n=87), education (19.5%; n=82) and a lack of evidence-based guidelines (16.7%; n=70). Less than half of the respondents reported practicing acupressure (12.8%; n=12), with most expressing uncertainty about the location of various acupoints and acupoints contraindicated before 37 weeks of gestation (61.7% to 87.2% across all acupoints). Findings from thematic analysis highlighted that increased maternal interest in natural approaches, creates a corresponding need for enhanced midwifery expertise in acupressure.

**CONCLUSIONS:**

The findings revealed midwives’ lack of adequate knowledge, education and training in using acupressure during the perinatal period. These themes mirrored the quantitative findings, reinforcing that interest in acupressure is present among Maltese midwives but is constrained by gaps in knowledge, training and policy support.

## INTRODUCTION

Acupressure, a component of traditional Chinese medicine with a history spanning thousands of years, is used to treat a range of ailments^1^. This therapy involves applying firm pressure to specific acupoints using fingers, thumbs, or specialized tools such as pens and wristbands^2^. From a therapeutic perspective, acupressure is believed to restore balance in the body’s natural functions, while physiologically it is thought to stimulate endorphin release, inhibit pain receptors, and trigger oxytocin secretion^1^.

In perinatal care, defined as the period from conception to six weeks postpartum^3^, evidence suggests that acupressure can reduce nausea and vomiting, relieve pain, induce labor, lessen anxiety and enhance breastmilk production^1^. Specific acupoints are associated with particular outcomes: stimulation of an acupoint on the inner wrist can alleviate morning sickness^1^, points above the ankle may improve uterine blood flow and oxytocin release, supporting effective contractions and shortening labor, and another acupoint located four fingers above the inner ankle may reduce postpartum afterpains through endorphin release^4^.

In midwifery, this practice is particularly relevant in making care less medicalized and increasing practices that support natural physiological processes. While complementary and alternative medicine (CAM) is increasingly being integrated into maternity care, acupressure practices by midwives remain inconsistent and often unsupported by formal training or clinical guidelines^5-7^. Yet, little is known about how confident midwives feel using CAM techniques like acupressure, particularly in the Maltese context.

Professionally, midwives are central to perinatal care and play a key role in advocating for physiological birth. By integrating acupressure, midwives may enhance women’s experiences and expand care options. This study aimed to explore midwives’ knowledge and views on the use of acupressure during the perinatal period while also identifying factors that influence their decision to use acupressure in clinical practice.

## METHODS

### Study design and data collection process

A cross-sectional approach was used in this study, where a self-administered online questionnaire was distributed via email. The population of 240 midwife members of the Malta Midwives Association (MMA) received details about the study. However, not all these midwives fulfilled the eligibility criteria to participate in the study, as not all were practicing midwives who were members of the MMA and working in maternity settings across the Maltese Islands; therefore, the number of eligible midwives was unknown. Retired or non-practicing members were excluded. As data collection progressed, questionnaires in paper format were distributed to eligible participants through an intermediary person to improve the response rate. In total, 94 answered questionnaires were returned from eligible participants.

Data collection was conducted between July and September 2024. Questionnaires were distributed electronically using Google forms and in paper format via the MMA office. Paper questionnaires were then returned anonymously in sealed boxes.

### Questionnaire development and pilot testing process

For this study, a self-designed questionnaire was developed by the researchers. The questionnaire was carefully designed to address key aspects aligned with the research objectives, drawing on the previously discussed literature. The questionnaire was divided into four sections, further detailed below:

*Section A: demographic data*

This section collected basic information about the respondents’ professional backgrounds.

*Section B: personal views*

This section explored midwives’ views on acupressure during the perinatal period. Participants were asked to indicate their level of agreement using Likert-type scale questions. These questions focused on statements related to the perceived benefits, safety and organizational support in the use of acupressure.

*Section C: knowledge of acupressure*

This section assessed the respondents’ knowledge and training on acupressure use during the perinatal period. It included questions about the definition of acupressure, its application, the respondents’ level of training, and whether they had practiced acupressure.

*Section D: influences for practice*

This section examined the factors that influence midwives’ confidence and practice of acupressure. Respondents indicated their confidence in discussing and using acupressure, factors influencing their decision to offer it, and reasons for any safety or efficacy concerns.

Since the tool used in this study needed to be validated, it was crucial to perform a stability assessment; therefore, the test-retest method was utilized to assess the reliability of the self-designed questionnaire. Fourteen participants who met the inclusion criteria were invited to pilot the questionnaire, with a one-week interval between the pre-test and post-test and all of them agreed to complete it. During the distribution of the pre-test questionnaire, each participant self-created a unique code to remember and later indicate on the post-test questionnaire. This coding system enabled the researchers to match each participant’s pre- and post-test questionnaires while maintaining anonymity. Additionally, participants in the pilot study were asked to fill in an evaluation form to provide their feedback on the clarity and relevance of the questions asked.

Face and content validity were identified as the most appropriate types of validity for this study and were subsequently assessed and established by an experienced academic midwife and researchers. This collaboration helped ensure that the questionnaire appeared reasonable and relevant to non-experts in the field. The pilot study confirmed clarity, feasibility, and stability of responses. No modifications to the questionnaire were necessary; therefore, the same questionnaire was used for the data collection among the 240 midwives, MMA members. The kappa (0.811; p=0.002) and Kendall tau tests (0.826; p=0.009) demonstrated a satisfactory test-retest result.

### Data analysis

Quantitative data were analyzed using IBM SPSS^®^ version 29. Descriptive statistics were generated for demographic data and survey responses. Inferential analyses, including Kruskal-Wallis and Friedman tests, were applied to examine associations between variables. Descriptive statistical analysis entails summarizing and characterizing data using techniques like frequency distribution and measures of variability (range, mean and standard deviation). Using frequency distribution analysis, categorical variables with one or more replies were graphically portrayed in tables or bar graphs according to the total number of participants. Responses to open-ended questions were analyzed thematically following Braun and Clarke’s framework, with themes derived inductively from the data^8^.

Likert scale questions were used to measure the level of agreement across various statements, to determine midwives’ views quantitatively. The Friedman test compares mean rating scores (Likert scale) between several related statements. These mean rating scores range from 1 to 5, where one corresponds to ‘strongly disagree’ and five corresponds to ‘strongly agree’.

The Kruskal-Wallis test was used to compare mean rating scores provided to a statement between groups of participants clustered by their work setting, years of experience, and level of education. These mean rating scores range from 1 to 5, where 1 corresponds to ‘strongly disagree’ and 5 corresponds to ‘strongly agree’.

## RESULTS

### Participant characteristics

Ninety-four practicing midwives and members of the Malta Midwives Association returned the completed questionnaires. Participants’ ages ranged from 24 to 63 years, with a mean age of 41 years. A wide range of professional experience was represented: the largest group had 1 to 10 years of practice (n=60; 63.8%), whereas midwives with 11 to 20 years of experience were the least represented (n=14; 14.9%). More than half of the participants (59.6%) reported holding a graduate qualification and most worked in the central delivery suite of the main public hospital in Malta (n=30; 31%), while the Assisted Reproductive Technology Clinic and Discharge Liaison Midwives were the least represented (n=2; 2.12%). Table 1 shows the participants’ demographic data.

**Table 1:** Demographic data of the study participants, midwives in the Malta Midwives Association (N=94)

*Characteristics*	*n*	*%*
**Place of work**		
Central delivery suite	27	28.7
Midwifery relieving pool	22	23.4
Obstetrics ward	21	22.3
Outpatients	14	14.9
Discharge liaison midwives	2	2.12
Neonatal pediatric intensive care unit	6	6.38
Maternity unit Gozo	3	3.19
**Education level in Midwifery**		
Diploma	1 1	11.7
Degree	56	59.6
Master’s	27	28.7
PhD		
**Total number of years working as a midwife**		
1–10	60	63.8
11–20	14	14.9
>21	20	21.3

### Midwives’ views on acupressure

Likert scale questions and multiple-choice questions were used to determine midwives’ views quantitatively. Table 2 illustrates the Friedman test used to compare mean values for the statements provided. The mean rating score provided to ‘Women should have the right to be informed about acupressure during the perinatal period’ and ‘All midwives should receive education on acupressure during their undergraduate curriculum’ (4.54) is the largest mean value, indicating the highest agreement. These are followed by ‘Women can be empowered with the use of acupressure as they can help in the administration of their own care’ (4.41), ‘I believe acupressure has the potential to alleviate pain during labor’ (4.32), and ‘Acupressure can be beneficial to all mothers in the maternity section’ (4.01). The mean rating score provided to ‘My hospital has guidelines/procedures on the use of acupressure during the perinatal period’ (1.55) is the smallest, indicating the lowest agreement. This is preceded by ‘The use of acupressure is painful’ (2.07), ‘I feel supported in my organization to recommend acupressure’ (2.19), and ‘I believe that the use of acupressure is a long process’ (2.51).

**Table 2:** Maltese midwives’ views on acupressure (N=94)

*Views*	*Rating score*	
	*Mean*	*SD*
Acupressure can be beneficial to all mothers in the maternity section.	4.01	0.898
Women should have the right to be informed about acupressure during the perinatal period.	4.54	0.799
Women can be empowered with the use of acupressure as they can help in the administration of their own care.	4.41	0.809
I feel supported in my organization to recommend acupressure.	2.19	1.040
My hospital has guidelines/procedures on the use of acupressure during the perinatal period.	1.55	0.798
Some acupressure points can be unsafe during pregnancy.	3.73	0.906
Acupressure is viewed as having a potential to increase choices for women with post-date pregnancies.	3.96	0.994
I believe acupressure has the potential to alleviate pain during labor.	4.32	0.907
The use of acupressure is painful.	2.07	1.019
I believe that acupressure may be attributed to psychological factors rather than physiological effects, suggesting a potential bias in wishful thinking.	2.81	1.008
I believe that the use of acupressure is a long process.	2.51	1.114
I believe that acupressure can help alleviate after pains post normal vaginal delivery.	3.73	0.832
I believe that acupressure can help increase milkflow production in the postpartum period.	3.77	0.944
All midwives should receive education on acupressure during their undergraduate curriculum.	4.54	0.713
There is inconclusive research on the use of acupressure during the perinatal period.	3.23	0.897

χ^2^(14)=716.499, p<0.001. Friedman test used to compare mean values for the statements provided in question 4, Likert scale question.

The mean rating score provided by NPICU staff to ‘I feel supported in my organization to recommend acupressure’ was higher among NPICU staff (3.50) compared to staff working on obstetrics wards (1.86) and discharge liaison midwives (1.00) (p=0.017). For the remaining statements, there was no significant discrepancy in the mean rating scores between the different work settings (Table 3).

**Table 3:** Reponses to the question ‘I feel supported in my organization to recommend acupressure’ among Maltese Midwives by work settings

*Item*	*Place of work*	*n*	*Rating score*	
			*Mean*	*SD*
**I feel supported in my organization to recommend acupressure.**	**Central delivery suite** (p=0.017)	26	2.08	1.129
	**Midwifery relieving pool**	22	2.14	0.834
	**Obstetrics**	21	1.86	0.727
	**Discharge liaison midwives**	2	1.00	0.000
	**Outpatients**	14	2.57	1.222
	**NPICU**	6	3.50	0.837

### Knowledge of acupressure

Most participants (91.5%; n=86) accurately defined acupressure as the application of firm pressure to stimulate an acupoint. However, a small proportion (7.4%; n=7) confused it with acupuncture, and one participant (1.1%) incorrectly associated it with the use of essential oils. The majority were unsure on ‘Acupoints that reduce blood pressure’ (62.1%; n=7), ‘Acupoints that reduce stress and anxiety’ (50%; n=60), ‘Acupoints helpful in managing the third stage of labor’ (72.1%; n =75), ‘Acupoints that improve breastmilk production (72.3%; n=73), ‘Acupoints which decrease labor afterpains’ (63.3%; n=62). Moreover, less than half of the respondents (48.9%; n=46) were able to correctly identify acupoints contraindicated before 37 weeks of gestation.

However, when asked which acupoints help in pain management during labor and postpartum period, most participants answered correctly, indicating BL32 (15.3%; n=27), GB21 (8.5%; n=15), LI4 (14.1%; n=25) and the Buttock point (12.4%; n=22), all of which help relieve tension, reduce pain and promote relaxation. Knowledge levels did not differ significantly by age or years of practice, 44.7% (n=42) of respondents who reported no formal education or training in acupressure. Among those who had received some form of training, self-directed learning through research or reading materials was the most common (20.2%, n=19), followed by workshop attendance without certification (18.1%; n=17). Only 2.1% (n=2) of participants had obtained a formal certification in acupressure training. Additionally, the majority (78.7%; n=74) reported never having practiced acupressure in maternity care. Among the minority who had (21.3%; n=20), labor was the most common stage of use (75.0%, n=15), followed by pregnancy (60.0%; n=12) and the postpartum period (15.0%; n=3). Several midwives commented in open-ended responses that although they had heard of acupressure, their understanding was limited and often based on informal sources rather than formal education or evidence-based literature.

### Factors influencing practice

When asked about prerequisites for practicing acupressure, data illustrated in Table 4, respondents reported that training and education were the most influential factors chosen by 87 participants (20.7%) and 82 participants (19.5%), respectively. Similarly, 70 participants (16.7%) indicated that the absence of evidence-based guidelines would discourage them from using acupressure. Moreover, 57 midwives (13.6%) also highlighted the importance of institutional support when practicing acupressure.

**Table 4:** Most commonly cited factors influencing midwives in Malta to offer acupressure (N=94)

*Item*	*Factors*	*n*	*%*
**What do you think influences midwives to offer acupressure?**	Education	82	19.5
	Training	87	20.7
	Support from organization	57	13.6
	Appropriate guidelines	70	16.7
	Support from colleagues	56	13.3
	Efficacy concerns	32	7.6
	Safety concerns	34	8.1
	Other	2	0.5

**Figure 1:**
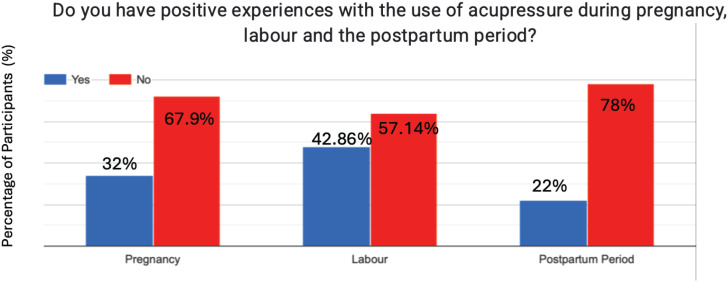
Illustrating midwives' personal use of acupressure during the perinatal period

Lastly, Figure 1 illustrates the personal use of acupressure among midwives during their own experiences of pregnancy, labor and the postpartum period. From this question, a large number of midwives reported no personal use of acupressure across all three periods, as evidenced by the predominance of ‘No’ responses. Contrastingly, the highest rate of use is observed during one’s own pregnancy, with 8.5% (n=8) of respondents reporting acupressure application. This is followed by a slight decrease during labor, where 5.3% (n=5) of midwives employed the technique. The postpartum period shows the lowest practice, with only 4.3% (n=4) of participants using acupressure.

### Qualitative findings

Analysis of open-ended responses provided a deeper view into midwives’ perspectives. These qualitative aspects of our questionnaire led to three overarching themes.

*Interest in complementary therapies (n=79)*

Midwives expressed a genuine interest in broadening their scope of practice by incorporating complementary approaches like acupressure. Several respondents noted that women are increasingly seeking natural, non-pharmacological methods to manage pregnancy and birth, and that midwives should be prepared to support these preferences. Moreover, many participants (n=30) reported attending courses, seminars, workshops and discussing with colleagues. One midwife highlighted:

*‘I try to stay updated by attending courses and workshops.’* (P12)

This engagement with structured learning experiences underscores their recognition of the value of expert-led instruction and the necessity to find time and stay updated with advancements in their field.

*Concerns about safety and competence (n=205)*

Despite this interest, midwives frequently voiced concerns about their ability to practice acupressure safely. Comments highlighted fear of causing harm if contraindicated points were used inadvertently, as well as apprehension about liability in the absence of clear institutional policies. Midwives expressed particular concern about the potential safety issues that could lead to maternal or fetal distress. In fact, a participant noted:

*‘If the practitioner is not confident in using acupressure, this may be a safety concern.’* (P3)

Moreover, participants indicated that they felt uninformed on the use of acupressure, and this uncertainty made them feel uncomfortable practicing it on mothers. Participants stating:

*‘I am not sure.’* (P20)

*‘I do not know.’* (P45)

shed light on their hesitation and expression of uncertainty.

*Need for education and guidelines (n=34)*

A strong theme was the call for formal education, clinical training and the establishment of national or institutional guidelines. Many midwives felt that acupressure should only be introduced into maternity care through guided programs. Participants emphasized the importance of structured guidelines and posters in the maternity setting for safe practice of acupressure, with one midwife suggesting:

*‘Clear hospital guidelines and posters around wards on acupoints could help safer and more confident practice.’* (P4)

These themes mirrored the quantitative findings, reinforcing that interest in acupressure is present among Maltese midwives but is constrained by gaps in knowledge, training, and policy support.

## DISCUSSION

This study explored midwives’ knowledge and views of acupressure during the perinatal period. While acupressure has been widely recognized as a complementary therapy with potential benefits during pregnancy, labor, and postpartum^1,4^, little is known about midwives’ knowledge and use of this technique in practice. Findings revealed that Maltese midwives were generally interested in acupressure but demonstrated knowledge gaps and limited confidence, with a lack of education and guidelines identified as major barriers to practice.

Comparable demographic distributions have been reported in international studies exploring midwives’ engagement with complementary and alternative medicine (CAM)^9-19^, particularly regarding practice settings, with most midwives working in central delivery suites or birth centres^9,16^. However, unlike earlier studies that predominantly featured midwives with more than a decade of clinical experience^5,13,15^, the majority of respondents in this study had between 1 and 10 years of experience, potentially reflecting a generational shift in openness toward complementary and alternative medicine (CAM).

Knowledge gaps were evident, particularly regarding contraindicated acupoints and safety considerations. Fewer than half of respondents could correctly identify acupoints as unsafe before 37 weeks’ gestation. This finding is especially noteworthy, as no prior studies directly assessed midwives’ knowledge of contraindicated acupoints in pregnancy. Nonetheless, these findings echo international research demonstrating that while midwives may be familiar with CAM modalities, their detailed knowledge is often limited^17^. The lack of confidence in identifying acupoints mirrors the results, which is also evident in Australian midwives who required specific training to integrate acupressure effectively into practice^14^. Despite these gaps, midwives expressed favorable perceptions of acupressure, recognizing its potential to alleviate labor pain, induce labor, ease afterpains and promote milk production. This aligns with previous studies suggesting midwives often view CAM modalities as supportive in enhancing maternal comfort and wellbeing during childbirth^14,16^.

Interestingly, knowledge did not vary significantly with years of experience, highlighting that professional exposure alone does not ensure competence in CAM. This supports the view that structured training, rather than experiential learning, is essential for safe practice^19^. Given that acupressure is promoted as a safe, low-cost, non-invasive therapy^1^, these knowledge gaps highlight an urgent need for educational interventions. Midwives in this study expressed notable interest in receiving further education and training, with over 90% of respondents supporting the integration of acupressure into professional development, and a similar proportion agreed that increased knowledge would enhance its clinical application. This demand echoes earlier research highlighting midwives’ enthusiasm for expanding their skillsets through continuing education, particularly in areas where formal training is limited^16,17^.

In order to address the identified knowledge gaps, it is recommended that acupressure be formally integrated into undergraduate midwifery curricula, combining theory with hands-on training^6^. For practicing midwives, structured continuing professional development (CPD) programs should be introduced, such as workshops and smallgroup sessions. Additionally, clear, evidence-based clinical guidelines should be developed through collaboration with professional bodies, including safety protocols and legal documentation practices to make midwives feel confident and safe when practicing acupressure^8,15^. Lastly, public education initiatives, such as parentcraft classes, are also advised to raise awareness among expectant mothers regarding the benefits of acupressure and its use in the perinatal period.

Most participants expressed positive attitudes towards acupressure, acknowledging its potential to support women in pregnancy, labor and postpartum. However, some were discouraged due to the lack of knowledge and safety concerns. This ambivalence reflects findings in other settings, where midwives valued CAM for broadening care options but were hesitant without formal training^19^. Midwives reported apprehension about inadvertently stimulating contraindicated points and the potential for adverse outcomes. These concerns are consistent with previous findings, where lack of knowledge was linked to reluctance to practice CAM^14,15^. Addressing these concerns requires structured training, supervised practice and clear guidelines to foster competence and confidence. The results suggest that midwives are willing to use acupressure if appropriate safeguards are in place. Midwives in this study also highlighted the need for more research on the use of acupressure throughout pregnancy and the postpartum period. This aligns with calls from previous studies for evidence-based research on CAM therapies before recommending them for use in clinical practice^11,15^. Future studies should include qualitative and mixed-methods approaches to capture both professional and maternal perspectives. Additionally, investigating barriers, facilitators and cost-effectiveness is essential to inform policy and practice while also evaluating the impact of different educational strategies on midwives’ competence. Additionally, randomized controlled trials (RCTs) are especially recommended to strengthen evidence-based practice, particularly in the under-researched postpartum phase^16^.

Participants highlighted growing interest among women in non-pharmacological methods, with midwives recognizing acupressure as a potential response to this demand. Previous studies confirm increasing maternal interest in CAM as part of holistic maternity care^10,19^. However, midwives noted that without professional knowledge, they risk being unable to meet women’s expectations. This gap between maternal demand and midwives’ preparedness could undermine woman-centered care. Integrating CAM education into midwifery curricula and continuous professional development would help ensure midwives are equipped to support maternal choices.

A consistent barrier identified was the absence of evidence-based guidelines. More than two-thirds of midwives felt unable to practice acupressure without clear professional frameworks. This reflects wider challenges in integrating CAM into conventional maternity systems, where a lack of policy direction hinders uptake^10^. Internationally, structured guidelines for CAM use in maternity care remain limited, leaving midwives reliant on informal sources or personal initiative. Developing context-specific guidelines would provide a framework for safe and standardized practice. Institutional support, through endorsement by professional bodies and inclusion in continuing professional development, would further enable integration.

### Strengths and limitations

A key strength of this study lies in its originality as the first Maltese investigation of midwives’ knowledge and views of acupressure to examine midwives’ knowledge and views of acupressure during the perinatal period, addressing a notable gap in the literature. The survey design with a combination of multiple-choice, closed-ended, and open-ended questions enabled us to gather comprehensive and meaningful data. Selection bias was avoided as participants were recruited through an intermediary, while anonymous data collection helped to avoid social desirability bias. Nonetheless, the use of self-reported questionnaires may have introduced response bias, with participants potentially overstating knowledge or interest. The cross-sectional design captures views at a single point in time, limiting the ability to assess changes over time or causality. Additionally, some correct responses in the knowledge section may have been answered by chance, potentially impacting the reliability of those results. Lastly, although a test–retest procedure was done, it was performed on a small sample size; therefore, future studies could evaluate the psychometric properties using a larger sample.

## CONCLUSIONS

The study emphasizes the importance of acupressure use during perinatal care and midwifery practice. Clinically, acupressure offers women a non-medicalized approach that may reduce reliance on pharmacological interventions while supporting natural physiological processes^1^. This aligns with current shifts towards holistic, women-centered models of care^4^. This study demonstrates that Maltese midwives are generally in favor of the use of acupressure as a potential addition to perinatal care but lack the necessary training, confidence and institutional support to practice it safely. Knowledge deficits were evident in identifying contraindications and locating acupoints. While maternal demand and midwives’ openness to complementary therapies may drive interest, the absence of guidelines, structured education, and professional support remains a major barrier.

## Data Availability

The data supporting this research are available from the authors on reasonable request.
